# Opportunities for research in molecular radiotherapy

**DOI:** 10.1259/bjr.20160921

**Published:** 2017-03

**Authors:** Glenn D Flux, Joe O'Sullivan, Mark N Gaze, Kevin M Prise

**Affiliations:** ^1^ Joint Department of Physics, Royal Marsden NHS Hospital and Institute of Cancer Research, Sutton, UK; ^2^ Centre for Cancer Research and Cell Biology, Queen's University, Belfast, UK; ^3^ Department of Oncology, NIHR University College London Hospitals Biomedical Research Centre, London, UK

## Abstract

Cancer has been treated with radiopharmaceuticals for 80 years. A recent National Cancer Research Institute report from the Clinical and Translational Radiotherapy Research Working Group reviews the current status of molecular radiotherapy and has highlighted the barriers to and opportunities for increased research activities. The report recommends a number of actions to promote this field, which in the dawning age of personalized medicine and theragnostics is of increasing importance, particularly with the clinical introduction of a range of new commercial radiotherapeutics at costs in line with those seen for conventional chemotherapeutics. These recommendations recognize the importance of a multidisciplinary approach to the development of molecular radiotherapy and the particular need for investment in radiopharmacies and personalized dosimetry. There are many areas to be investigated including adaptive treatment planning, the use of radiosensitizers and translational radiation biology. Progress in these areas will result in significant patient benefit and more cost-effective use of increasingly expensive therapeutic radiopharmaceuticals. A concerted effort from the community, from funding bodies and from health service providers is now needed to address the scientific and logistical changes necessary to realize the potential offered by this currently underused treatment modality.

## INTRODUCTION

Radioiodine therapy was conceived in 1936 following a meeting between the clinician Saul Hertz and the physicist Karl Compton.^1^ Initial treatments were performed with radiation measurements and dosimetric analysis. On the 80th anniversary of this auspicious meeting, a National Cancer Research Institute (NCRI) report, produced by the Clinical and Translational Radiotherapy Research Working Group (CTRad), has highlighted the dramatic changes that are under way, the huge challenges faced and the great potential for development of molecular radiotherapy (MRT).^2^ In 2003, a similar report for external beam radiotherapy (EBRT) highlighted the need for investment in research that led to the formation of the CTRad.^3^

CTRad was established in 2009 by the NCRI with a clear mission to promote clinical and translational research in radiation oncology. Members include clinical oncologists and clinicians from other disciplines, radiographers, physicists and other scientists. CTRad has four work streams covering the science base, early and late phase clinical trials, and physics and new technologies. CTRad activities include support for the development of clinical trials; advice on radiotherapy quality assurance (QA) in clinical trials; and partnership with the pharmaceutical industry to investigate drug–radiation interactions. The achievements of CTRad in its first quinquennium, and its vision for the future, are well documented.^4^

The report entitled “CTRad : Identifying opportunities to promote progress in molecular radiotherapy research in the UK” was the result of multiprofessional input from a number of key individuals and professional bodies interested in molecular oncology research. The aim of the report included identification of the strengths and weaknesses of MRT research in the UK and identification of the barriers to research. The report recognizes the importance of MRT as a cancer therapy and acknowledges that there needs to be a significant increase in clinical and translational research activity in the field.

The report was compiled using data from a CTRad MRT questionnaire, sent to 58 National Health Service nuclear medicine departments, as well as interviews with UK MRT professionals.

There are 10 recommendations in total, the key recommendations being:

– the establishment of a forum through which the MRT community can better engage with research funders and potential collaborators
– funding to increase the number of appropriately trained MRT professionals with protected research time
– increased investment in radiopharmacies
– routine dosimetry-based treatment planning for MRT
– the establishment of a national QA group to deliver full QA in MRT trials.

The report introduces remarkable potential for development, in many cases along similar lines as are seen for EBRT, which will result in improved patient benefit. Avenues for exploration include the following.

### Radiosensitizers and concomitant administration of chemotherapeutics

The targeting agent is simply a vector to deliver the radionuclide to the tumour cells. Cytotoxicity results from the emitted radiation causing single- and double-strand breaks in the nuclear DNA. As cells have some intrinsic ability to repair potentially lethal damage, concomitant administration of drugs which may interfere with this may act as radiosensitizers.^5^ There is laboratory evidence of synergistic interactions between MRT and both topoisomerase 1 inhibitors (for example topotecan)^6^ and poly(ADP-ribose) polymerase inhibitors (for example olaparib).^7^ There is as yet no clinical evidence that combinations of DNA repairing inhibitors and MRT are truly an improvement over MRT alone, but combinations are feasible to deliver.^8^ Clinical and pre-clinical research is essential to evaluate the true benefit of these combinations.

### Translational radiation biology

The recent developments in MRT have highlighted the need for high-quality radiation biology research to maximize its future translational potential. For radionuclide exposures, the absorbed dose deposition and subsequent biological response is constrained by two key physical parameters—the decay rate of the isotope (and hence the absorbed dose rate) and the type of radiation decay, *i.e.* the radiation quality (or linear energy transfer). Understanding the relationship of this to individual patient treatments from a dosimetric perspective needs both pre-clinical and clinical research. From a biological perspective, there is now rapid progression from an understanding of a direct DNA damage response-driven mechanism to more complex signalling-driven mechanisms where the triggering of systemic responses, including activation of immune responses, has the potential to deliver significant patient benefits in the metastatic setting.^9,10^ To translate studies through to clinical application, understanding is needed of the underpinning mechanisms of MRT exposures. This requires fundamental radiation biology research in relevant pre-clinical models which mimic the clinical situation, including individualized tumour responses. In addition, trained research personnel with the expertise to deliver high-quality pre-clinical studies in advanced tumour models are urgently required.

### Adaptive treatment planning

It has been demonstrated extensively that while the range of absorbed doses delivered to different patients varies widely, sequential treatments for several therapy procedures, particularly those given within 4–6 weeks, result in similar biokinetics.^11,12^ This introduces the strong potential to perform adaptive treatment planning, whereby activities are calculated to deliver a prescribed absorbed dose. Clinical trials are urgently needed to determine whether such an approach is feasible.

### Combined molecular radiotherapy and external beam radiotherapy

For many diseases in which MRT may have a role, there is also a place for EBRT. An example is neuroblastoma, where mIBG therapy for refractory disease may be followed by EBRT to the primary tumour site, and possibly also residual distant metastatic sites. For optimal tumour kill, and to avoid excess late effects on normal tissues, it is essential to know not just the physical but also the biological effect of both modalities individually and combined.^13^ This important area is poorly understood and should be a priority for future clinical and pre-clinical research programmes.

## DISCUSSION

The lack of development in MRT relative to the progression seen in EBRT is understandable and is not limited to the UK. A small number of patients are treated with this modality, usually with rare cancers and often only with palliative intent. An exception is the treatment of thyroid cancer with radioiodine, which in patients at low risk has proven so successful that treatments have remained essentially unchanged in the 80 years since it was first used. This has helped perpetuate the myth of the “magic bullet”.

It is no exaggeration to say that MRT is now entering a new era that will cause significant changes at all levels. A number of new innovative radiotherapeutics are entering the clinic at costs similar to those for conventional chemotherapeutics, necessitating health economic evaluation at a national level. Revision of the risks to patients from planned and unplanned treatments is of increasing importance in Europe, with the introduction of a directive due to be enacted in February 2018 concerned with basic safety standards.^14^

MRT is increasingly regarded as a form of radiotherapy rather than chemotherapy, whereby activities should be administered according to personalized treatment planning, based on the absorbed doses delivered to target volumes and to normal tissues. Such a sea change offers unprecedented opportunities for a multidisciplinary approach to personalized treatment, but will require a major revision of infrastructure, resourcing and management that must involve all related disciplines and organizations.

The recent NCRI/CTRad report clearly articulates a range of research activity and barriers for MRT in the UK and lays out strategic priorities that will be of significant benefit to the research community.

## FUNDING

We acknowledge NHS funding to the NIHR Biomedical Research Centre at RMH and ICR.
